# BODIPY Dye Derivative for Irreversible Fluorescent Labeling of Eukaryotic Cells and Their Simultaneous Cytometric Analysis

**DOI:** 10.32607/actanaturae.26879

**Published:** 2023

**Authors:** A. Yu. Frolova, S. V. Kutyakov, V. I. Martynov, S. M. Deyev, A. A. Pakhomov

**Affiliations:** M.M. Shemyakin and Yu.A. Ovchinnikov Institute of Bioorganic Chemistry of the Russian Academy of Sciences, Moscow, 117997 Russian Federation

**Keywords:** fluorescence, chromophore, cytometry, flow cytofluorimetry, BODIPY, cell labeling, cell analysis

## Abstract

In this work, we synthesized a green fluorescent dye derivative, 1,3,5,7-tetramethyl-BODIPY, with a heptyl substituent at the 8-position. The obtained highly hydrophobic compound was able to rapidly and irreversibly bind to eukaryotic cells. Incubation of cells with the dye over different periods of time or at different concentrations allowed us to control the degree of cell labeling and the level of fluorescence. This made it possible to modulate the fluorescence level of different eukaryotic cell cultures and then distinguish them by their level of fluorescence signal in the green channel in cytometric experiments. The labeled cells can be combined and further analyzed in the same test tube under identical conditions using the channels in which the dye does not fluoresce. This approach has been tested on a number of tumor cell cultures containing the HER2 receptor on their surface. The representation of the receptor in these cells was analyzed in one test tube in one run using a HER2-specific ligand based on the hybrid protein DARPin9_29-mCherry, which fluoresces in the red region of the spectrum.

## INTRODUCTION

Flow cytofluorimetry is a technique widely used for studying the functioning of living systems, developing new drugs, and in medicine for sample analysis and selection of treatment strategies. The method is based on the labeling of cells with fluorescent dyes, often conjugated with proteins targeted to various surface markers (e.g., antibodies), which allows one to phenotype the cells in a population [1, 2]. It is often necessary to compare cell cultures with each other during analysis, for example, to compare cell parameters with control samples. In this case, the samples are analyzed in parallel under identical conditions. However, it is impossible to provide completely identical conditions when preparing cell samples from experiment to experiment because of the inherent errors in the sample preparation due to pipetting, as well as the potential influence of the human factor.

In this study, we elaborated an approach that allows one to simultaneously analyze several cell cultures in one test tube, even when they initially do not differ in the parameters detected cytometrically. For this purpose, different cell samples are pre-stained with a nonspecific dye so that each analyzed culture has a different fluorescence intensity in one of the detection channels of the cytometer. This is possible by treating the cells with a lipophilic dye that binds nonspecifically to cell membrane structures at different times or different concentrations.

## EXPERIMENTAL

**Synthesis of 8-heptyl-4,4-difluoro-1,3,5,7- tetramethyl-4-bora-3a,4a-diazaindacene (BDP-C7)**

Octanoyl chloride (1 mL, 10 mmol) and 2,4-dimethylpyrrole (650 mg, 4 mmol) were successively dissolved in dry dichloromethane (50 mL) pre-flushed with argon. The mixture was stirred for 3 h at room temperature under argon atmosphere. Triethylamine (3 mL, 22 mmol) was then added, and after 15 min at 0°C, boron trifluoride etherate (3 mL, 24 mmol) was added portion-wise (as three portions). The mixture was further stirred at 0°C for 3 h. After completion of the reaction, the resulting mixture was passed through a short column filled with a silica gel using toluene as an eluent. The solvent was evaporated, and the reaction product was separated by column chromatography on a silica gel using toluene as an eluent. The yield was 558 mg. 1H-NMR (400 MHz, CDCl3): δ 0.89 (t, 3H, C*H*3CH_2_), 1.31 (m, 6H, CH_3_C*H*2C*H*2 C*H*2), 1.48 (m, 2H, CCH_2_CH_2_C*H*2), 1.63 (m, 2H, CCH_2_C*H*2), 2.41 (s, 6H, CH_3_), 2.51 (s, 6H, CH_3_), 2.92 (t, 2H, CC*H*2), 6.05 (s, 2H, CH). 13C-NMR (101 MHz, CDCl3): δ 14.0, 14.4, 16.3, 22.6, 28.5, 29.0, 30.4, 31.7, 31.9, 76.7, 77.0, 77.3, 121.5, 131.4, 140.3, 146.7, 153.7. 19F-NMR (376 MHz, CDCl3): δ 146.66 (q, J 19F-11B = 32.3 Hz).

**Spectrophotometry**

The absorption and fluorescence emission spectra were measured using a Cary50 Bio spectrophotometer (Varian) and a Cary Eclipse spectrofluorometer (Varian), respectively. The fluorescence quantum yield was measured using a homologous derivative of BODIPY, 8-decene-1,3,5,7-tetramethyl-BODIPY [3, 4, 5], as a standard (the quantum yield of the standard in DMSO was considered to be 0.99).

**Cell culture**

SKBR3 human breast adenocarcinoma cells overexpressing the HER2 tumor marker [6], modified EMT6/P mouse mammary carcinoma cells overexpressing HER2 (EMT-HER2) [7], and HeLa human cervical cancer cells with a normal HER2 expression level [8] were cultured in DMEM (Gibco, Thermo Fisher Scientific, Inc, USA) supplemented with 10% fetal bovine serum (FBS, Gibco, Thermo Fisher Scientific, Inc.), 100 units/mL penicillin, 0.1 mg/mL streptomycin, and 0.25 μg/mL amphotericin (Gibco, Thermo Fisher Scientific, Inc.) at 37°C and 5% CO_2_ in a humidified atmosphere. The growth medium was renewed every 2 days. The Versene solution (PBS and 0.02% EDTA) was used during culturing.

**Flow cytofluorimetry**

SKBR3, EMT-HER2, and HeLa cells were harvested with the Versene solution and precipitated at 125 g for 5 min; the supernatant was removed, and PBS containing 10% FBS was added to the cells to a concentration of 5 × 10^3^ cells/μL. Then, 100 μL of a solution of the **BDP-C7 **dye at a given concentration in PBS containing 1% DMSO was added to 3 μL of cell suspension. The cells were incubated at room temperature for the specified time, sedimented at 500 g for 30 s, and the supernatant was removed. Next, either 100 μL of PBS was added to the cells and cytometric analysis was performed, or a PBS solution containing the 1 μM DARPin-mCherry protein was added, incubated for 5 min and after sedimentation, removal of the protein solution and addition of 100 μL of PBS, a cytometric analysis was performed. A Novocyte 3000 VYB flow cytofluorimeter (ACEA Biosciences, USA) was used for the cell analysis. Green detection channel (FITC): laser excitation at 488 nm, emission detection through a 530/30 nm light filter; red detection channel (PE-Texas-Red): laser excitation at 561 nm, emission detection through a 615/20 nm light filter. Before analyzing the fluorescence level of the cells, events corresponding to living cells were first selected (gating in A-FSC / A-SSC channels), and then events corresponding to non-aggregated cells were selected (gating in A-FSC / H-FSC channels) [9].

**Cell viability study**

HeLa cells cultured in complete DMEM were seeded in a 96-well plate (104 cells/well) and grown overnight. Prior to testing, the culture medium was removed and 100 μL of a fresh medium containing **BDP-C7 **at the specified concentration and 1% DMSO was added. The **BDP-C7 **substance was tested in a concentrations ranging from 33 nM to 20 μM in three repeats. Sample solutions were prepared by serial 2.5-fold dilution of the concentrated sample. The culture medium containing 1% DMSO was added to the control cells. After overnight incubation, liquid was withdrawn, and 100 μL of 3-(4,5-dimethylthiazol- 2-yl)-2,5- diphenyltetrazolium bromide (MTT) solution at a concentration of 5 mg/mL in a serum-free culture medium was added to each well and the cells were further incubated at 37°C for 3 h. The supernatant was then removed, and 100 μL of DMSO was added to dissolve the formazan crystals. The optical density was measured at 570 and 640 nm using an Infinite M1000 Pro plate reader (Tecan, Austria).

## RESULTS AND DISCUSSION

**Fig. 1:**
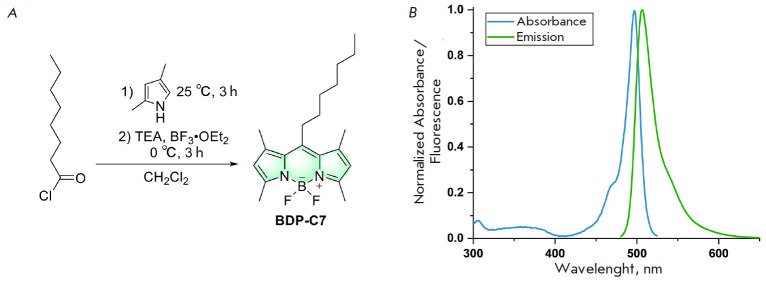
Synthesis of the **BDP-C7 **dye (*A*) and its absorption and fluorescence emission spectra in DMSO (*B*)

A derivative of 1,3,5,7-tetramethyl-BODIPY containing a heptyl substituent at the position 8 (**BDP-C7**, *Fig. 1A*) was chosen as a dye to demonstrate the applicability of the proposed approach. BODIPY derivatives are characterized by high brightness and photostability; they possess narrow fluorescence excitation and emission bands, allowing them to minimally “interfere” with other dyes [10, 11, 12, 13, 14]. Examples of the use of BODIPY, including in cytometric tasks, have been described [15, 16, 17]. The methyl groups in **BDP-C7 **protect the chromophore from interactions with the external environment, while the heptyl substituent increases the hydrophobicity of the dye and facilitates its irreversible binding to cell membrane structures.** BDP-C7 **was synthesized starting from octanoyl chloride according to the protocol used previously for homologous compounds [3, 18, 19] (*Fig. 1A*).

When dissolved in DMSO, the dye exhibited narrow excitation and fluorescence emission bands, with the maxima of light absorption and fluorescence emission at 497 and 507 nm, respectively (*Fig. 1B*). The extinction coefficient was 87300 M-1cm-1, and the fluorescence quantum yield was 99%. Thus, **BDP-C7 **is a bright fluorescent dye and is ideally suited for detection in the green channel of most fluorimetric instruments with laser excitation at 488 nm and detection in the 495–525 nm range.

**Fig. 2:**
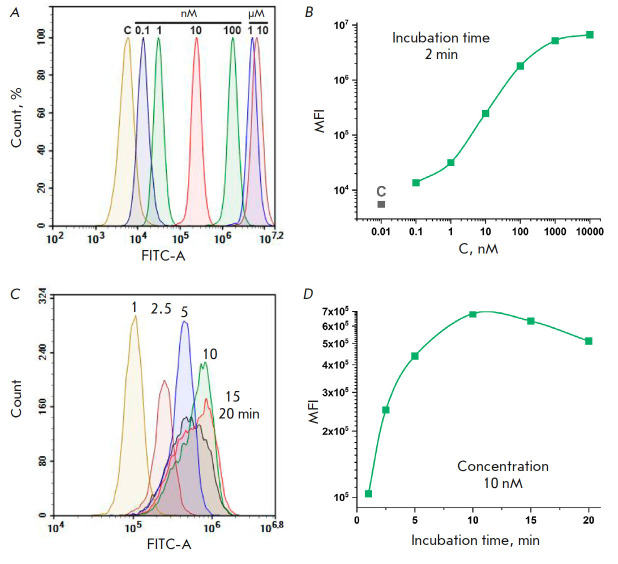
Cytometric analysis of HeLa cells exposed to the **BDP-C7** dye for 2 min at concentrations ranging from 0.1 nM to 10 μM (*A, B*) and at 10 nM for 1–20 min (*C, D*). C – control cells not subjected to dye treatment. The histograms of the intensities in the green fluorescence channel with excitation at 488 nm and emission detection at 530/30 nm (*A, C*) and the median fluorescence intensity (MFI) values of the cell populations (*B, D*) are shown

To test the staining of eukaryotic cells with the** BDP-C7 **dye, we used the HeLa cell culture, which is widely used in routine cell experiments. First, the cells were stained at different concentrations of** BDP-C7 **for a short period of time. The cells were incubated for 2 min in PBS containing **BDP-C7 **at a given concentration and 1% DMSO. After incubation, the unbound dye was washed off, and the cells were analyzed on a flow cytofluorimeter (*Fig. 2A,B*). *Figure 2A* indicates that the concentration of 1 nM is sufficient to distinguish the treated cells from the control, untreated cells. At 1 μM of the dye, there is probably almost maximum saturation of the cell with the dye; treatment with higher concentrations increases the fluorescence level only slightly. One can see that the samples treated with 1, 10, 100 nM, and 10 μM of the **BDP-C7 **dye are well distinguishable from each other and from the control. Thus, by staining HeLa cells with the **BDP-C7 **dye, we successfully obtained five populations that were well distinguishable in one detection channel. The number of such populations can be increased to at least six due to the region around 10 nM.

Treatment of the cells with the **BDP-C7 **dye at the same concentration but during different time periods (*Fig. 2B,D*) also yielded cells with different levels of green fluorescence, but this effect was not so significant. *Figure 2B* demonstrates that the level of cell fluorescence rises by less than an order of magnitude as the incubation time is increased from 1 to 10 min, while broadening of the peak due to the shoulder in the low-intensity region is observed upon incubation for 10 min. As the incubation time is increased to 15 and 20 min, in addition to peak broadening, the median cell fluorescence decreases. This is most likely to be caused by changes in cell morphology during the 10–20 min of incubation under suboptimal conditions (1% DMSO in PBS). At other concentrations of **BDP-C7**, the effect was similar (data not shown). Therefore, incubation of cells with the dye for more than 5 min makes no practical sense because of the changes occurring in the cells under unfavorable conditions and the resulting broadening of peaks; moreover, treatment with the dye at different concentrations for a short time period allows one to achieve a difference in the fluorescence signal of the cells of several orders of magnitude (*Fig. 2A,B*).

**Fig. 3:**
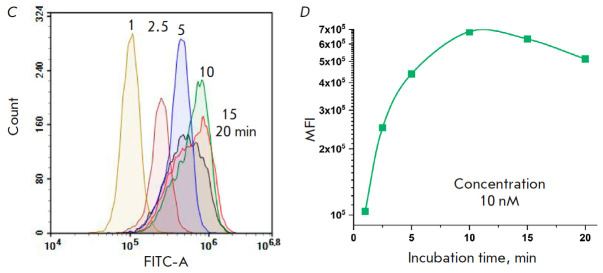
Analysis of the **BDP-C7 **dye washout from the cells. Cytometric analysis of HeLa cells exposed to the dye for 2 min at concentrations of 1, 10 and 100 nM. Cells after washing to remove the unbound dye and incubation in PBS buffer for a given time. Histograms of the intensities in the green fluorescence channel (*A*) and the median fluorescence intensity (MFI) of the cell population (*B*) at different incubation times after washing are shown

Next, we tested whether dye washout and fluorescence signal changes occur after **BDP-C7 **binding to the cells. For this purpose, after washing off the unbound dye, the cells were incubated in PBS in different time intervals and then analyzed on a flow cytofluorimeter (*Fig. 3A*). In all the samples tested, the level of the fluorescent signal remained virtually unchanged with time (*Fig. 3*). We did not test times longer than 30 min, since this time period is usually sufficient for the manipulations required for a cytofluorimetric analysis.

**Fig. 4:**
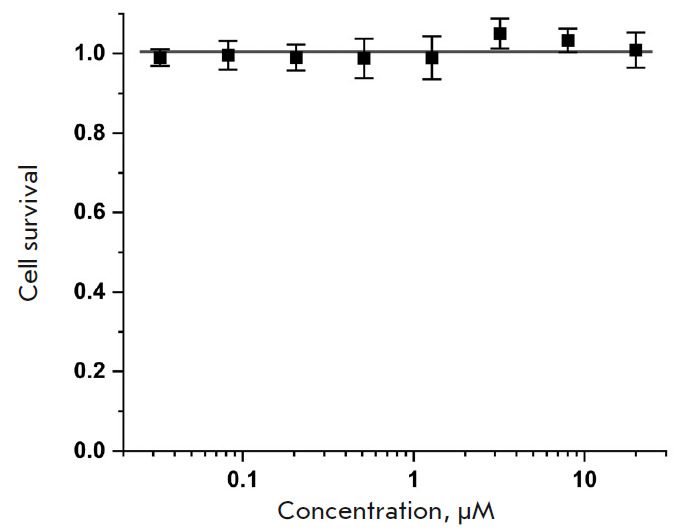
Cytotoxicity of the **BDP-C7 **dye as measured by MTT assay on HeLa cells

We also tested whether the dye could exhibit cytotoxicity at the concentrations used. Cytotoxicity was tested using the standard MTT assay at dye concentrations of up to 20 μM (*Fig. 4*). **BDP-C7** showed no toxicity in the entire tested concentrations range.

The applicability of the approach consisting in labeling cell cultures by staining with the green fluorescent dye **BDP-C7 **at different concentrations was tested on HeLa, SKBR3, and EMT-HER2 cell cultures. These cells differ from each other in the expression level of the surface tumor marker, human epidermal growth factor receptor 2 (HER2). It is estimated that HeLa cells contain a small (normal) amount of HER2 on the surface [8], whereas the receptor is overexpressed in SKBR3 [6] and artificially derived EMT-HER2 [7]. The representativity of the receptor on the cell surface can be tested using HER2-targeted fluorescent antibodies [20, 21], as well as ligands based on designed ankyrin repeat proteins (DARPins). We used the fusion protein DARPin9_29-mCherry [22], where DARPin9_29 is a targeting protein that efficiently binds to HER2, and mCherry is a red fluorescent protein that provides fluorescence of the construct in the red region of the visible spectrum.

**Fig. 5:**
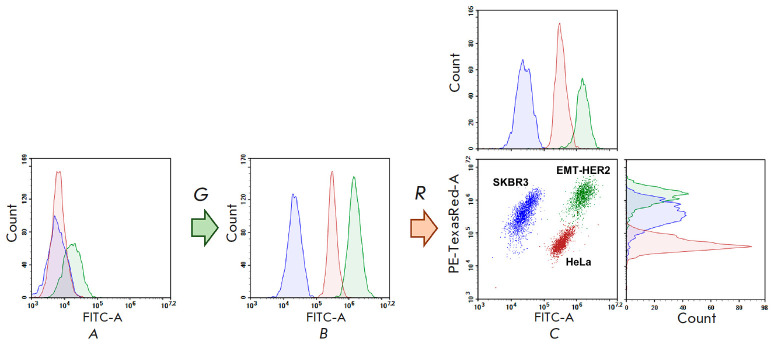
Cytometric analysis of the SKBR3, HeLa, and EMT-HER2 cells, untreated (*A*), treated separately first with the green **BDP-C7 **dye at concentrations of 1, 10, and 100 nM, respectively (*B*), then with red ligand to the HER2 tumor marker (DARPin9_29-mCherry, *C*). An overlay of the results obtained for each cell line in parallel experiments. Data for HeLa are shown in red; for SKBR3, in blue; and for EMT-HER2, in green

We first stained the cell cultures separately with the **BDP-C7 **dye and DARPin9_29-mCherry to estimate the level of HER2 representation on different cultures. In order to distinguish the cell cultures from each other in the green channel, the SKBR3, HeLa, and EMT-HER2 cells were treated for 2 min with a PBS solution containing 1% DMSO and **BDP-C7** at concentrations of 1, 10 and 100 nM, respectively (*Fig. 5*). One can see from the overlay of cell fluorescence histograms in the green channel that, after staining with **BDP-C7**, the cultures are fairly well differentiated from each other in terms of the fluorescence signal (*Fig. 5B*). After staining of the cells with the DARPin9_29-mCherry protein for the HER2 tumor marker (*Fig. 5C*), the HeLa culture differed well from EMT-HER2 in the red channel, whereas the SKBR3 cells used by us had an intermediate fluorescence value.

**Fig. 6:**
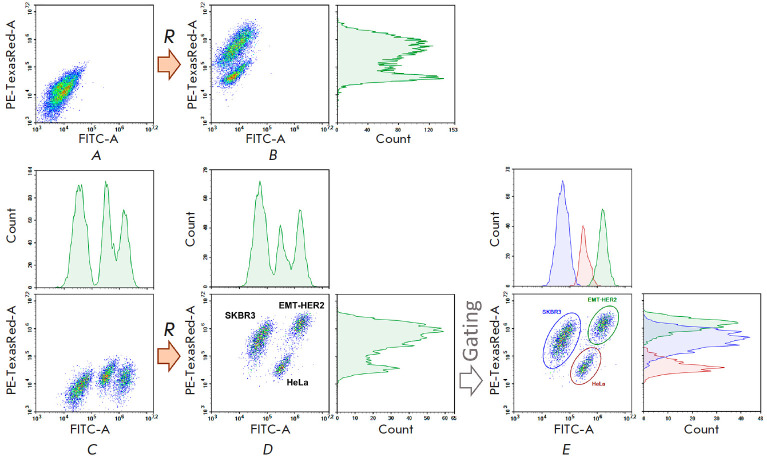
Cytometric analysis of the HeLa, SKBR3, and EMT-HER2 cells mixed together before staining (*A*) and then treated with a red ligand to the HER2 tumor marker (DARPin9_29-mCherry, *B*). In (*C*), the cells were pre-stained with the green dye **BDP-C7 **at different concentrations, washed and mixed in a single test tube, and then treated with DARPin9_29-mCherry (*D*). (*E*) – identification of individual cell populations and analysis of their fluorescence in the green (FITC) and red (PE-Texas Red) channels

To compare the representation of the HER2 tumor marker on the analyzed cultures under identical conditions, we mixed SKBR3, HeLa, and EMTHER2 cells in one test tube and then treated them with the DARPin9_29-mCherry protein (*Fig. 6*). One can see that when the cells were not pretreated with** BDP-C7**, after staining, the HeLa culture containing a small amount of HER2 on its surface is partially separated from the cells that overexpress the receptor, but it is impossible to distinguish SKBR3 and EMT-HER2 from each other (*Fig. 6A,B*).

If the cells are labeled with **BDP-C7 **before mixing (*Fig. 6C*), then after staining with the DARPin9_29- mCherry protein, three clearly distinguishable cell populations can be observed in the dot plot showing the level of cell fluorescence in the green and red channels (*Fig. 6D*). By gating these cell populations, one can determine their belonging to a particular culture according to the level of the fluorescent signal in the green channel (*Fig. 6E*), since each culture was labeled with a green fluorescent dye at different concentrations. In the red channel, the fluorescence level of each population can be quantified to assess the representation of the HER2 tumor marker.

We compared the results of the analysis of the HER2 representation in the tested cultures according to the data obtained in three experiments for each cell culture separately and the data obtained in one test tube after labeling the cells with **BDP-C7** (*Table 1*). The results were found to almost fully match. According to the data obtained, in the SKBR3 cell line, the HER2 tumor marker was an order of magnitude more abundant than in HeLa, and HER2 representation in EMT-HER2 cells was additionally 3.5-fold higher.

**Table 1:** Comparison of the results of the cytometric analysis of HER2 representation on the EMT-HER2, SKBR3 and HeLa tumor cells obtained in three independent experiments, as well as in an experiment with cell cultures pre-labeled with the BDP-C7 dye and mixed in a single test tube

Cell culture	Parallel experiments		In a single test tube	
	MFI^*^	HPCV^**^	MFI^*^	HPCV^**^
EMT-HER2	1 410 051	61.08%	1 380 511	61.64%
SKBR3	411 140	86.96%	410 552	100.83%
HeLa	46 678	123.57%	39 616	38.15%

^*^MFI – median fluorescence intensity in the red channel (PE–Texas Red).

^**^HPCV – half-peak coefficient of variation.

HPCV = FWHM / (2.36 × X) × 100%, where FWHM is the full width at half maximum, X is the mean of the dataset.

The developed approach allows one to mix several cell populations in one sample and analyze them in a single test tube under completely identical conditions. Therefore, the potential artifacts associated with a variation of the concentrations of the substances acting on the cell, caused by pipetting errors during sample preparation or carelessness by the experimenter, are minimized. In addition, this approach allows one to reduce the consumption of ligands to the analyzed cell receptors, since the assay is performed in a single test tube rather than in different ones for each cell culture. This may be relevant if the ligand is commercially unavailable or the quality of the ligand varies between batches. Our approach allows one to save time, since multiple samples can be analyzed in a single run. However, time must first be spent to fluorescently label the cells with the dye at different concentrations and adjust the concentrations, so that the cells become clearly distinguishable in the fluorescence channel of the dye. In addition, using extra dye “takes” one channel of detection.

## CONCLUSION

In the present work, we have described an approach that allows one to perform a cytometric analysis of different cell cultures in a single test tube: i.e., under completely identical conditions. In this case, it is possible to analyze cells that do not initially differ in any “marker” receptors. Using the **BDP-C7 **dye, the cells can be labeled in the green channel and the level of the fluorescent signal desired in each individual experiment can be adjusted.

Instead of the **BDP-C7 **used in this work, other fluorescent dyes can theoretically be used. But it should be kept in mind that the dye needs to be chemically stable and bright enough to provide good contrast, be highly hydrophobic to nonspecifically bind well to the cells, and be nontoxic at the concentrations used.** BDP-C7 **perfectly meets all of these requirements, while dyes containing double bonds and extended aromatic systems can be easily oxidized in cells and undergo spectral transformations due to this fact. Many polar dyes cannot accumulate well in the membrane structures of the cell and can be eliminated from the cell over time.

## Abbreviations

BDP-C7: 8-heptyl-1,3,5,7-tetramethyl-BODIPY
BODIPY: 4,4-difluoro-4-bora-3a,4a-diaza- s-indacene
DARPin: Designed Ankyrin Repeat Protein
HER2: human epidermal growth factor receptor 2
